# Non-prosthetic peri-implant fracture of both forearm bones

**DOI:** 10.1093/jscr/rjad300

**Published:** 2023-08-08

**Authors:** Jose Antonio Prieto Meré, Sergi Barrera-Ochoa, Dorka Liburd-Hernández, Jésica P Presas

**Affiliations:** icatMA Hand and Microsurgery Unit, ICATME, Hospital Universitari Quiron-Dexeus, Barcelona, Spain; Orthopedic and Traumatology Department, Instituto Guatemalteco de Seguridad Social (IGSS), 13 avenida Calzada San Juan, 01057 Guatemala city, Guatemala; icatMA Hand and Microsurgery Unit, ICATME, Hospital Universitari Quiron-Dexeus, Barcelona, Spain; icatMA Hand and Microsurgery Unit, ICATME, Hospital Universitari Quiron-Dexeus, Barcelona, Spain; Department of Orthopedics, Hospital Central del Ministerio de Defensa, Santo Domingo, República Dominicana; Department of Orthopedics, Hospital Interzonal General de Agudos Dr Luis Güemes Haedo, Haedo, Buenos Aires, Argentina

**Keywords:** Forearm, Non-prosthetic peri-implant Fracture, Peri-implant Fracture, Non-prosthetic peri-implant

## Abstract

Peri-implant fractures occur in association with an implant that was used to treat a previous injury. Peri-implant fractures are considered relatively ‘new’ fractures for which there is no accepted classification system in practice. Treatment is difficult due to altered anatomy, the presence of orthopedic implants and phenomena such as stress shielding, osteopenia when not in use, and fracture remodeling. We present the case of a young man who presented to the emergency room after a sports accident with a successful previous osteosynthesis and a new deformity of the forearm.

## INTRODUCTION

Non-prosthetics peri-implant fracture (NPPIF) is a fracture in a bone with an existing non-prosthetic implant such as an extramedullary plate and screws or an intramedullary nail. NPPIFs are an under-reported entity [1]. Studies reporting upper limb NPPIFs are focused on the effect of removal of implants as this appears to be associated with a significantly higher risk of re-fracture compared to if they are retained.

## CASE REPORT

**Figure 1:**
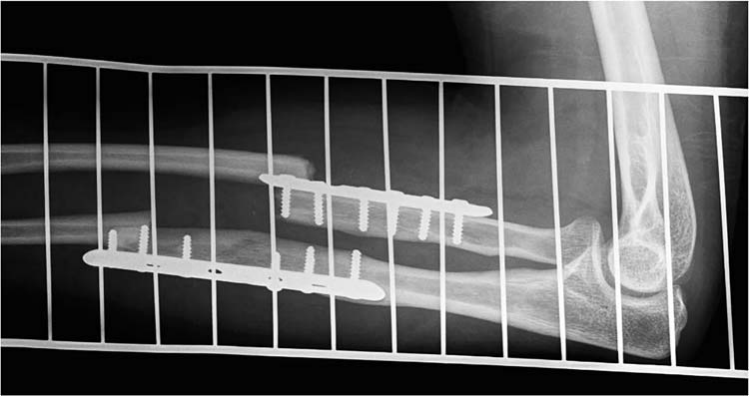
Type P1A fracture—well healed original fracture with NPPIF of both forearm bones distal to the tip of the implant.

**Figure 2:**
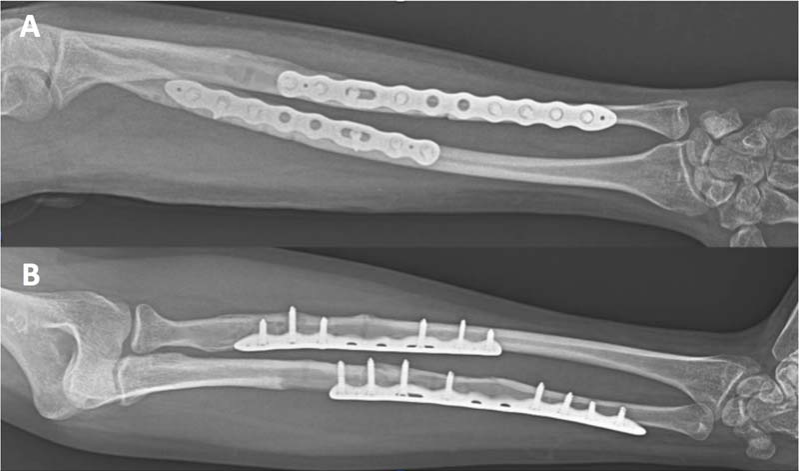
Internal fixation of both NPPIFs with a longer plate that bridges the original fracture zone and the new NPPIF. Final follow-up at 36 months after surgery, anterior–posterior X-ray.

A 32-year-old male was referred to the emergency room after a sport accident. Fourteen months previously, he successfully underwent osteosynthesis with two 3.2 mm stainless steel locking plate (Trimed Elbow Forearm System™, CA, USA) for a radial (six-holes) and ulnar (seven-holes) shaft fractures after a sport accident. On admission, X-rays revealed a NPPIF of both forearm bones (Fig. 1), classified as P1A type [2]. The fracture was just distal to the edge of the last screw of both plates. The patient underwent both hardware removal, reduction of the fractures and fixation using two 3.5 mm longer titanium dynamic compression plate for the radial (eight holes) and ulnar (10 holes) shaft fracture (Stryker Corporation Kalmazoo, MI, USA). After that, the patient was referred for physical therapy and rehabilitation. A total of 3 months after surgery, complete bone healing was observed (Fig. 2). At final follow-up, 36-months after surgery, the patient’s elbow extension and flexion was from 0° to 130°, wrist pronation and supination was from 90° to 75°, and wrist flexion and extension were 75° and 70° were completely painless. His grip strength was 93% that of the opposite hand.

## DISCUSSION

The few studies that have reported on upper limb NPPIFs have focused on the effects of removing implants, as this appears to be associated with a significantly higher risk of re-fracture than if they are retained [3]. Decision making regarding implant removal depends on the opinion of the surgeon who has to decide between two possible situations, re-fracture in implant-removed bone and NPPF in implant-retained bone. It can be speculated that re-fracture in implant-retained bone is protected because of the higher degree of stress shielding provided by the compression plate. However, in type 1 NPPIFs, as in this case, the implant has acted mechanically as a stress amplifier, predisposing the bone to further fracture [2, 4]. Rigid fixation has been repeatedly shown to result in a transient increase in porosity and thinning of cortical bone within the first few months. [3, 4]. In this case, we hypothesized that the first fixation with stainless steel locking plates was too rigid, favoring NPPFs, and therefore the second osteosynthesis with titanium dynamic compression plates was more elastic. Although routine implant removal is not recommended in adults with forearm shaft fractures treated with compression plates [3], we think this case should be considered to evaluate implant removal after bone healing or to perform a more dynamic fixation initially. NPPIF are an interesting but little studied entity. This is a very rare NPPIF on both forearm bones. In the largest series of NPPIF in the literature, only one patient had a similar fracture pattern to our patient [2]. However, surgical management is challenging because in the remaining NPPIFs, the type 1 fracture can be treated by replacing the implant with a longer implant that bridges the original fracture zone and the new NPPIF [1, 2]. With the increasing use of osteosynthesis and an aging population, the number of such fractures will undoubtedly increase and represent an important clinical challenge. For this reason, surgeons should be aware of options to treat this complication.

## CONFLICT OF INTEREST STATEMENT

None declared.

## FUNDING

None.
